# Development of an evidence-based knowledge translation intervention to promote behavioral change in cerebral palsy diagnosis

**DOI:** 10.3389/fpubh.2026.1753404

**Published:** 2026-03-16

**Authors:** Vivian W. Y. Wong, Stephanie M. Glegg, Olivia Scoten, Chetna Jetha, Anamaria Richardson, Jill G. Zwicker, Mor Cohen-Eilig, Ram A. Mishaal

**Affiliations:** 1 Rehabilitation Sciences Graduate Program, Faculty of Medicine, University of British Columbia, Vancouver, BC, Canada; 2 BC Children's Hospital Research Institute, Vancouver, BC, Canada; 3 Department of Occupational Science and Occupational Therapy, Faculty of Medicine, University of British Columbia, Vancouver, BC, Canada; 4 Department of Pediatrics, Faculty of Medicine, University of British Columbia, Vancouver, BC, Canada; 5 Royal London Hospital, Barts Health NHS Trust, London, United Kingdom; 6 Sunny Hill Health Center at BC Children's Hospital, Vancouver, BC, Canada

**Keywords:** cerebral palsy, clinical practice guidelines, diagnosis, implementation, knowledge translation, practice behaviors, practice change

## Abstract

This article describes the development of an intervention designed to change community pediatricians' practice of diagnosing cerebral palsy (CP) for low-risk term-born infants and children in British Columbia (BC), Canada. A multidisciplinary team co-designed a knowledge translation (KT) intervention to facilitate detection and diagnosis of CP by pediatricians using the Theoretical Domains Framework, the Behavior Change Wheel, and AIMD Framework. A list of desired change objectives was determined based on current CP diagnostic guidelines. Pre-identified barriers and facilitators were analyzed and subsequently mapped to evidence-based change strategies. Strategies selected for implementation were then expanded to specify the intervention's aims, ingredients, mechanisms, and delivery. The resulting theory-informed KT intervention was comprised of accredited continuing medical education (CME) workshops targeting community pediatricians to adopt six change objectives to reach the goal of earlier CP diagnosis and improved health outcomes for children with CP in BC. Through an iterative process, the intervention was tailored to maximize adoption of targeted objectives by addressing pediatricians' pre-identified barriers while accounting for local health systems context. Evaluation of participants' intentions to change, adoption of change objectives, and acceptability of intervention delivery will be reported in a subsequent publication. This novel KT intervention involves community-based pediatricians as target participants and enhances traditional CME offerings by incorporating implementation science theoretical frameworks to change practice behaviors.

## 1 Introduction

Cerebral palsy (CP) is the most common physical disability in children (1, 2). As with other neurodevelopmental conditions, diagnosis is generally recommended as early as possible (3–5). Early diagnosis is critical to timely CP-specific early interventions and supports, to prevent medical complications (6) and to alleviate prolonged caregiver distress (7, 8). Failure to provide intervention during the first years of life, when neuroplasticity is highest, presents a major lost opportunity to optimize growth and development for children with CP (9). Randomized clinical trials increasingly demonstrate how CP-specific early interventions can maximize neuroplasticity to provide improved motor and cognitive outcomes (9–12). CP can be diagnosed accurately in infants before 12 months, or in some high-risk cases, as early as 3 months with the use of international early diagnosis guidelines and recommended assessments (9). Half of infants with CP are considered to be high-risk and have medical risk factors early on in the newborn period, enabling prompt detection and screening. The other half of the CP population typically are term-born with no remarkable medical history until later in life when developmental warning signs appear (3, 9).

Major shifts in clinical practice are required to enable early detection and diagnosis for all children at risk for CP. Researchers are advised to provide more information on how to implement evidence-based guidelines in practice, beyond recommendations based largely on academic research (13). Implementation science (IS), the study of ‘methods to promote the systematic uptake of research findings and other evidence-based practice into routine practice' [(14), p.1], is well-positioned to support new efforts to change CP diagnostic practices.

Despite availability of published care pathways for diagnosis (15, 16), children still receive a CP diagnosis in high-income countries between 12–24 months (9) and in low- and middle-income countries, the median age of diagnosis was 3 years old (17). The mean age of diagnosis in Canada is approximately 19 months (18). Diagnosis is even later at 25 months on average in the province of British Columbia (BC), as per a recent analysis of data from the Canadian Cerebral Palsy Registry (19). The concern is particularly heightened for those term-born infants and children with little or no early discernible risk factors. Assessment and diagnosis typically happen much later in community settings for this low-to-moderate risk subpopulation, compared to high-risk preterm infants who are monitored by neonatal follow-up clinics or other pediatric subspecialists (9). This subpopulation also tends to have a higher degree of motor abilities, or functioning at lower Gross Motor Function Classification System (GMFCS) levels (20, 21). This difference is apparent in an analysis of the Canadian Cerebral Palsy Registry which found that a child in BC with CP functioning at GMFCS level I (“walks without restrictions”) (22) was diagnosed on average 2 years later than one at GMFCS V (“Transported in a manual wheelchair”) (19, 22). Parents and caregivers have also raised concerns about delays and complex diagnostic timelines, well after the first warning signs have been reported to a healthcare professional (8, 23). This scenario in BC exemplifies an archetype example of the clinical guidelines problem, where barriers to their usage are well documented and strategies to increase systematic uptake have long been studied (24–27).

Physicians have long hesitated to refer, or delayed and refrained from diagnosing CP (3, 28). Well-documented reasons include a lack of understanding of the clinical definition, fear of misdiagnosis, and a preference to ‘wait and see' to first rule out other diagnoses (28–30). This traditional ‘wait and see' approach is harmful, especially when early intervention is not provided while being monitored, as understood now from the large body of evidence on the impact of CP-specific early interventions during the first 2 years of life. Often, physicians delay communicating the diagnosis in hopes of finding an alternative less severe disorder or that the child will “grow out of” the motor delays (3). A 2018 survey of BC pediatricians revealed that a significant proportion were not diagnosing CP despite believing that early diagnosis was important and falls within their scope of practice (31). An interdisciplinary team was formed in Vancouver, BC to address this significant knowledge-practice gap, defined as the gap between established scientific knowledge and its application in routine healthcare practice (32) and improve health outcomes for children with CP and their families using IS methodology. The goal of this implementation study was to decrease the age of diagnosis for children with CP in BC through provider adoption of current CP diagnostic guidelines at the community level. The primary objective was to co-design a knowledge translation (KT) intervention aimed to increase pediatricians' clinical comfort in detecting and diagnosing CP. The secondary objective was to evaluate the intervention's effectiveness to change practice behaviors and its acceptability by participants. This article focuses on objective one; objective two methods and findings will be described elsewhere.

## 2 Method

The study team was comprised of medical experts in developmental pediatrics, pediatric neurology, IS, community pediatrics, occupational therapy, hospital-based patient and family engagement adviser, continuing medical education specialists, a graduate research trainee, and a medical student. The majority of the study team also were members of a team at BC Children's Hospital leading the CP Early Diagnosis Clinic for high-risk infants and its clinical leaders were responsible for the study as part of a larger provincial initiative. The graduate trainee and IS expert led the study design and implementation processes, the CP Early Diagnosis Clinic team's developmental pediatricians and pediatric therapists curated the education content for the intervention, and the continuing medical education specialist provided consultative, marketing, and logistical support to organize the intervention activities. The intervention design incorporated diverse information sources: targeted participants' input on diagnostic practices and learning needs from surveys and regional champions, expert opinions from clinicians and caregivers of children/young adults with CP, relevant theoretical frameworks for behavior change, and evidence of physician practice change strategies from the literature. Limited availability due to competing schedules resulted in necessary concurrent working sessions between the graduate trainee and the CP Early Diagnosis Clinic staff, the community pediatrician and the IS expert. Through a highly iterative process, the graduate trainee consolidated feedback into the intervention design repeatedly and coordinated a meeting with the full study team to determine final intervention objectives, mechanisms of change, ingredients, and delivery mode. Engagement during the design process can be described as ‘collaborate' according to the IAP2 Spectrum of Public Participation, whereby advice and recommendations were incorporated to the maximum extent where possible (33).

To enable a tailored intervention design for target participants, the team first conducted a follow-up survey with BC pediatricians in 2022 using the Theoretical Domains Framework (TDF) (34) to identify barriers and facilitators to diagnosing CP in clinical practice, classified as determinants of behaviors. The survey method was chosen to elicit as many voices as possible from target participants from a variety of practice locations and settings. Pediatricians were recruited online via the University of British Columbia's Department of Pediatrics, the BC Pediatric Society, and the Rural Coordination Center of BC. Responses were coded as barriers or facilitators according to the TDF by the graduate trainee and medical student. Disagreements were resolved by the IS expert on the team. Key barriers were chosen to be tailored into the intervention after analysis of data using frequency counts, expert review, and subsequent discussion around feasibility of addressing barriers. These key barriers related to the TDF domains of knowledge, skills, beliefs about capabilities, beliefs about consequences, social and professional role/identity, and environmental context and resources (Table 1). Knowledge gaps included not knowing the proportion of children with CP that are born pre-term vs. full-term, and the age at which predicting the severity of CP is most accurate. Skills gaps pertained to the use of recommended assessments for diagnosis, interpretation of their results, and recognizing motor type, topography, and severity of CP. Beliefs about capabilities barriers included uncertainty about ruling out other causes of motor delay, making a false positive diagnosis, and diagnosing suspected cases of CP involving children under two, and/or those who present with low tone (35). Echoing results from the 2018 study, facilitators included agreement on the importance of early diagnosis and recognizing CP diagnosis as part of their professional role (31).

**Table 1:** Pediatricians' barriers of diagnosing CP.

**Examples from survey responses**	**Barrier (TDF)**	**COM-B**
• “I don't know whether a diagnosis can be made before 12 months.” •“I feel it is very important that CP diagnosis should not be made until a child is at least 2 years.”	Knowledge	Capability
• “I am not familiar or trained to use any assessments.” •“I have difficulty recognizing early motor type, topography, and severity of CP.”	Skills	Capability
• “The family does not believe or respect my opinion, asks for further referral.” •“I prefer to refer to a specific specialist for diagnosis; I do not think it is my role to provide the diagnosis.”	Social/professional role	Motivation
• “Not sure what the relevance of diagnosis is if child is already receiving supports.” •“It is not important to provide a diagnosis of CP since it will not change the child's outcome or if the child already has access to therapy or supports.”	Beliefs about consequences	Motivation
• “I do not feel comfortable diagnosing when I am unable to classify a GMFCS.” •“I'm not always confident I can rule out other causes of motor deficits.” •“I am not sure of the next steps after making a diagnosis.”	Beliefs about capabilities	Motivation
• “Limited appointment time to do assessments” •“Lack of imaging services to support diagnosis” •“Time required or lack of time to learn about diagnosing CP”	Environmental context and resources	Opportunity

CP, cerebral palsy; GMFCS, gross motor function classification system (22); TDF, theoretical domains framework (34); COM-B, capability, opportunity, motivation – behavior framework (37).

Survey results enabled tailoring of the intervention design by establishing six TDF domains as pre-specified barriers (Table 1) that will be addressed by the intervention and facilitate practice change. Tailoring is defined as using change interventions and strategies that integrate prospectively identified barriers to change (36). Barriers were then mapped to recommended evidence-based change functions or mechanisms based on their linkages to underlying behavioral motivations, as per the Capability, Opportunity, Motivation, Behavior (COM-B) Model (part of the Behavior Change Wheel) (37). Subsequently, the Aims, Ingredients, Mechanism, and Delivery (AIMD) Framework was used to further plan and organize intervention specifications. These meta-frameworks are amalgamations of existing frameworks and theories that help probe possible determinants of change and identify evidence-based change functions to incorporate into a well-defined, theory-driven intervention (38).

All possible change mechanisms were considered for implementation feasibility and for their potential to support participants in reaching the desired change objectives. Six change objectives, aligned with best practice guidelines for CP diagnosis (9, 39, 40) were determined as the intervention's anticipated outcomes. These outcomes were finalized by consensus between the study team's two clinical experts and community pediatrician after two rounds of discussion. All but two of the recommended BCW change functions (Environmental Restructuring and Restrictions) were chosen and incorporated into the intervention. Table 2 outlines the intervention logic using the AIMD Framework that lists the mechanisms used to theoretically achieve each change objective, along with corresponding details of key active ingredients from the literature, and selected delivery modes (Table 2). Ultimately, change mechanisms and the delivery mode of CME were chosen based on feasibility factors including study budget, the study team's time availability and resources, and the comfort and ability of team members to deliver the mechanisms successfully. This selection process was performed informally through a team discussion. The functions of environmental restructuring and restrictions were not included in the intervention as they required means and systems-level authority beyond the study team's reach. During the deliberation and selection of delivery modes, the study team also accounted for available resources and capacity, the likelihood of impact on targeted participants, and learning format preferences from the 2022 survey.

**Table 2:** Proposed intervention.

	**AIMD framework**			
**Change objective**	**Aims**	**Ingredients**	**Mechanisms**	**Delivery**
1. Increase in knowledge of the evidence and benefits of early diagnosis of CP	• To accept that diagnosing CP falls within their roles/responsibilities • To understand and accept why a diagnosis of CP is important for children and families • To confirm role and expertise with families	• Content is authored and delivered by regional CP experts • Comprehensive and concise evidence summaries • Family testimonials - parents share stories of their journey toward answers and diagnosis	Education Persuasion Modeling	• In-person dinner presentations • Live online workshops
2. Take action upon detection of risk factors	• To transfer knowledge of clinical/developmental risk factors	• Content is authored and delivered by regional CP experts • Call out key risk factors and urge participants to not wait to take action	Education Training	
3. Perform assessment(s) promptly	• To identify what appropriate assessments are required for diagnosis • To prompt immediate assessment or refer for assessment(s) • To train in required assessments (if needed)	• Content is authored and delivered by regional CP experts (BCCH/SHHC) • General pediatrician (representing population of intervention participants) demonstrate how it is possible to do the assessments within standard appointment times	Education Training Enablement Persuasion Modeling	
4. Increase of knowledge, skills, and confidence in application for clinical decision-making	• To gain confidence in applying clinical knowledge to diagnose CP (interpreting and synthesizing)	• Regional CP experts and general pediatrician(s) demonstrate applying criteria to diagnosis • General pediatrician (representing population of intervention participants) demonstrate how it is possible to use guidelines to change practices and provide early diagnosis	Education Training Enablement Persuasion Modeling	
5. Communicate diagnosis to family	• To communicate, educate, and support families • To transfer knowledge of most common parent questions about CP	• Regional CP experts and general pediatrician(s) demonstrate applying criteria to diagnosis • Demonstration of a well-planned and compassionate diagnosis conversation between provider and family • Parent speakers to demonstrate impact of a well-delivered diagnosis and assessment experience	Education Modeling Persuasion	
6. Refer to early intervention and supports.	• To share what current supports and resources are available • To enable referrals to available resources for families	• Recommendation of evidence-based early interventions from regional CP experts	Education Training Enablement Persuasion Modeling	

CP, cerebral palsy; AIMD, Aims, ingredients, mechanisms, and delivery (65); BCCH/SHHC, BC children's hospital/sunny hill health center for children.

The team and the local Continuing Medical Education (CME) provider [University of British Columbia, Faculty of Medicine, Division of Continuing Professional Development (UBC CPD)] collectively reviewed education format options, costs, logistics involved, and their relative potential for integrating the BCW mechanisms to reach the intervention's change objectives, using the APEASE criteria (Acceptability, Practicability, Effectiveness, Affordability, Side-effects, and Equity) (41). The following section describes the outcomes of these processes—namely, the intervention plan developed to support uptake of the clinical guidelines by community pediatricians.

## 3 Results

### 3.1 Target population

As the intervention was expected to facilitate clinical practice changes, the CP diagnosis challenge was defined in behavioral terms at the individual provider level. Targeted participants of this intervention were general pediatricians who practice in community-based settings across BC, either full- or part-time. Many pediatricians working in more rural and remote regions practice both in community and perform more specialized roles in regional or tertiary care, such as neonatal intensive care units, or intermediate nurseries. Those who practice exclusively in hospital settings were not eligible to participate. Community pediatricians were selected as target participants based on Child Health BC's Tiers of Service framework, which outlines how pediatric health services are coordinated in the province (42). Under ‘child-focused development and habilitation service', community-level pediatricians are responsible for screening and/or diagnostic assessment to diagnose common neuromotor impairments, such as CP (43). Community pediatricians receive a significant volume of referrals of children with signs of developmental delay or other neuromotor concerns.

### 3.2 Expected outcomes

The proposed intervention was expected to yield six change objectives for participating community pediatricians:

1. Increase in knowledge of the evidence and benefits of early diagnosis of CP;
2. Take action immediately upon detection of risk factors for CP;
3. Perform assessments promptly;
4. Increase in knowledge, skills, and confidence in clinical decision-making related to making the diagnosis;
5. Communicate diagnosis to family;
6. Refer to early intervention and supports.

### 3.3 Intervention design

The intervention was organized and marketed for potential participants as accredited CME in partnership with UBC CPD. Categorized under the banner of ‘educational meetings' by the Cochrane Effective Practice and Organization of Care group, CME is a health professional behavior change strategy and can encompass formats, such as conferences, workshops, or courses, where information is shared (44). Systematic reviews over the years concluded that CME does improve physician performance and has a more reliably positive impact on physician performance than on patient health outcomes (45). Compared to no intervention, CME as the single intervention or the main component of a multi-faceted intervention have slightly improved health provider compliance rates with desired practices (45). As an incentive to participate, pediatricians could receive up to 1.5 Maintenance of Certification (MOC) Section 1 study credits for completion of any intervention activity (46). A set of five CME dinner presentations and four online workshops incorporated the change functions of education, training, enablement, persuasion, and modeling to achieve the targeted change objectives. According to the BCW's model of behaviors, these functions theoretically work to influence the underlying determinants most responsible for change by addressing six pre-identified TDF barriers (Table 3) (37). Outlined in Table 4 are the selected BCW functions and how they were operationalized as change strategies in the implementation plan.

**Table 3:** Intervention mapping.

**Change objective**	**Barriers (TDF)**	**COM-B (BCW)**	^*^BCW mechanisms
1. Increase in knowledge of the evidence and benefits of early diagnosis of CP	Knowledge	Psychological capability	Education Persuasion Modeling
	Beliefs about consequences	Reflective motivation	
	Social/professional role	Social opportunity	
2. Take action upon detection of risk factors	Knowledge	Psychological capability	Education Training
	Skills	Physical capability	
3. Perform assessment(s) promptly	Knowledge Skills	Psychological capability	Education Training Enablement Persuasion Modeling
	Beliefs about capabilities	Physical capability	
	Beliefs about consequences	Reflective motivation	
	Environmental context and resources	Physical opportunity	
4. Increase of knowledge, skills and confidence in application for clinical decision-making	Knowledge	Psychological capability	Education Training Enablement Persuasion Modeling
	Skills	Physical capability	
	Beliefs about capabilities	Reflective motivation	
	Environmental context and resources	Physical opportunity	
5. Communicate diagnosis to family	Knowledge	Psychological capability	Education Modeling Persuasion
	Beliefs about consequences	Reflective motivation	
	Social/professional role	Social opportunity	
6. Refer to early intervention and supports	Knowledge	Psychological capability	Education Training Enablement Persuasion Modeling
	Skills	Physical capability	
	Environmental context and resources	Physical opportunity	
	Beliefs about consequences	Reflective motivation	

CP, cerebral palsy; BCW, behavior change wheel (37); TDF, theoretical domains framework (34).

^*^BCW mechanisms apply to all barriers listed.

**Table 4:** Implementation strategies to target selected TDF barriers.

^*^BCW mechanism	**Barrier (TDF)**	**Intervention details**
Education	• Knowledge • Beliefs about capabilities • Social and Professional role/identity • Beliefs about consequences	Educational content in both delivery formats covered the key identified knowledge gaps on: (1) the evidence and benefits of early diagnosis of CP; (2) etiology, typography, and classification of CP; (3) evidence-based warning signs for CP; (4) clinical criteria required for CP diagnosis; (5) associated developmental delays; and (6) co-occurring conditions common in CP. Workshop facilitators integrated content into structured lists, interactive case studies, and handouts throughout presentations to allow pediatricians to detect CP and diagnose with confidence using evidence-informed guidelines. (Meets objectives 1-6) Creation and dissemination of two major resources: the BC Cerebral Palsy Community Diagnostic Care Pathway and BC Cerebral Palsy Community Diagnostic Toolkit. The Pathway is a brief bedside decision-making support tool created in recognition of community pediatricians' gap in knowledge of CP's clinical red flags, diagnostic criteria, and the required physical and neurological assessments to obtain them. The pathway was adapted from international guidelines for early detection of CP and supplements new guidance for diagnosis in this low-to-moderate risk subpopulation who have no other neonatal risk factors, such as specifying scenarios that necessitate MRI, and options for troubleshooting complex cases. To complement the Pathway, a 28-page toolkit was created to serve as an in-depth comprehensive guide for step-by-step CP diagnosis, clinical considerations for different assessments, and post-diagnosis support. (Meets objective 1-6)
Training	• Skills • Environmental context/resources	To address the identified key skills gaps from the barriers and facilitators survey, training focused on: (1) taking a CP-specific approach during consultation appointments with infants and children who exhibit developmental delays; (2) conducting required assessments (neurological and physical exams, full developmental history) for clinical synthesis and decision-making, including practical tips to conduct assessments (e.g., testing of specific reflexes); (3) recognizing findings that indicate clinical status of ‘high-risk of CP'; and (4) how to communicate a diagnosis directly to caregivers using family-centered language. Videos of facilitator performing key sections of the Hammersmith Infant Neurological Exam (HINE) provides an introduction and in-depth skill-building to one of the most recommended assessments for CP diagnosis. (9, 59) (Meets objective 3) Case studies used during workshops facilitated practice of identifying clinical warning signs in history and parent reports, selecting the appropriate assessments needed, and evaluating assessment results to determine if criteria of CP is fulfilled to make a diagnosis. (Meets objective 2, 3, 4)
Enablement	• Environmental context/resources	Physician-to-physician consult service – an academic detailing (40) session is offered to community pediatricians for one-on-one guidance on CP diagnosis or to assist with tone management decision-making during a 20-minute phone call or videoconference with pediatric neuromotor specialists based at BC Children's Hospital. This on-demand service supports enabling of best practices by directly addressing pediatricians' clinical challenges or questions related to CP diagnosis. (Meets objectives 1-6) Provision of practical tips – Medical Services Plan (MSP) billing codes (60). Participants were informed and reminded that specific billing codes are designated for longer appointments required for assessments, repeat assessments, interpretation of results or, and family discussion. This enables proper time management and compensation for the recommended diagnostic steps. (Meets objective 3-5) • 00511 Complex Consultation: 1-hour appointment for full neurological exam • 00545 Pediatric Case Conference: For time spent on the Physician-to-Physician Consult Service • 00554 Extended subsequent office visits: For follow-ups and to enable sufficient time for post-diagnosis CP management discussions with family
Persuasion	• Beliefs about capabilities • Social and Professional role/identity • Beliefs about consequences	The intervention was designed to highlight the urgency of implementing CP diagnosis guidelines by presenting the disparities experienced by children with CP in BC with delayed diagnostic timelines, in contrast to those in other jurisdictions. A caregiver video reinforced why CP diagnosis is needed via a video testimonial, sharing her personal experience and perspective about why a diagnosis is important, what it facilitates, and the harmful impact of delayed or withheld diagnosis for her child over the last five years. (Meets objective 1, 3) Another caregiver video reinforced the value of using best communication practices for diagnosis disclosure in a way that meets the needs of caregivers. A video testimonial was used to reiterate the missed developmental potential from her child's absent diagnosis and the emotional impact of a poorly communicated diagnosis from the healthcare provider. (Meets objective 1, 5) Throughout workshops, facilitators repeatedly emphasized the key message about how community pediatricians play a critical role for children at risk of CP; they are knowledgeable and capable to diagnose CP. (Meets objectives 1-4)
Modeling	• Beliefs about capabilities • Social and Professional role/identity • Beliefs about consequences	A community pediatrician speaker demonstrated how CP diagnosis is not only possible, but feasible in BC community settings by drawing from their own clinical experiences. Facilitators shared what they currently do in their early diagnosis clinic as regional developmental medicine specialists and shared best practices that are aligned with current evidence-based guidelines (9). (Supports objective 2-6)

CP, cerebral palsy; BCW, behavior change wheel (37); TDF, theoretical domains framework (34).

^*^BCW mechanisms apply to all barriers listed.

As recommended by UBC CPD, the delivery mode of dinner presentations remains a traditional and popular CME format for BC physicians. Five in-person events were held in major urban centers, where majority of community pediatricians' practices are based in BC. While the live online workshops targeted the same change objectives as the in-person events, educational content was spread over four sessions to make virtual participation as engaging as possible. These online workshops integrated dedicated time for interactions with facilitators and other interactive activities, such as polls, annotated training videos, and question-and-answer periods. Facilitators frequently checked in with participants about presentation pacing and reminded online participants to ask questions on camera or submit questions via the chat box at any time during the presentation. An additional moderator from the study team monitored questions submitted and supported any technical issues participants experienced at all online workshops. Breakout groups for discussing the case study were also smaller in size and led by additional developmental pediatric experts recruited for online workshops. Participants could register in one to four sessions. Parts of the educational content were repeated to provide multiple opportunities for participation and to reinforce key messages aligned with change objectives for participants who chose to attend more than one event. Other parts were adapted for ‘beginner' and ‘advanced' participants, recognizing pediatricians' varying levels of familiarity with CP. The advanced workshop included an expedited review of CP basics and clinical warning signs, and more in-depth discussions on interpreting assessment results and clinical reasoning for diagnosis. All intervention activities were offered free of charge.

### 3.4 Outcome measures

Surveys at baseline, post-intervention, and 3-month follow-up assessed pre-intervention practices, intention to adopt, and actual attainment of the targeted change objectives, respectively. As the intervention was conceptualized as CME activities to eligible community pediatricians, survey questions used an adapted version of the CPD REACTION Questionnaire to measure participants' intention to adopt change objectives and whether adoption of the behaviors took place post-intervention. This questionnaire is a validated theory-informed tool that evaluates the impact of CPD activities on clinicians' behavioral intentions (47). The tool demonstrates adequate validity and reliability, with acceptable to good levels of internal consistency (Cronbach's coefficients 0.77–085) (47). While these psychometric properties support measuring changes in behavioral intention, the questionnaire has little predictive validity for actual behavior change. Adaptations were made by the IS expert and streamlined lengthy standardized questions in the questionnaire regarding current practice behaviors, and levels of knowledge, skills, and beliefs about capabilities were used across each timepoint to determine to what extent the intervention resolved the pre-identified barriers to diagnosing CP in community settings. Additional questions in the follow-up survey were designed to measure if practice behavior changes had taken place. Adoption of change objectives was considered to be true if participants indicated they had performed any of the three practice behaviors (objectives 3, 5, 6) or an increase in level of skills, knowledge, and confidence was observed between before and after intervention (objectives 1, 2, 4). A pre-survey was sent a week prior to each workshop, with multiple reminders and the opportunity to complete the survey onsite if participants had not done so prior to the workshop. The post-intervention survey, sent to participants immediately and with reminders up to 2 weeks after the workshop, included evaluation questions related to satisfaction with and delivery of the intervention. The follow-up survey was sent 3 months after the workshop date and asked participants to share any changes related to CP diagnosis since the intervention and if practice barriers remained. Invitations to complete this follow-up continued to be sent up to 6 months after the workshop date. Because participants had the flexibility to sign up for one, some, or all intervention activities, all surveys asked about number of attended workshops to measure intervention dosage. Any participant interested in registering for a subsequent workshop was required to indicate which previous session they had already attended before starting the pre-, post-intervention, and follow-up surveys. The requirement to complete the surveys was seen as a potential barrier to participation, therefore the evaluation component was not mandatory. Results of this intervention will be summarized elsewhere using the Standards for Reporting Implementation Studies (StaRI) (48) and incorporate recommendations from Lengnick-Hall et al. (49) for improved implementation outcomes reporting.

## 4 Discussion

Calls for the explicit use of theory in KT and IS studies have increased as these fields expand, particularly with respect to what factors drive clinician behavior change (38, 50–54). Specifically, past interventions that attempted to address the evidence-to-practice gap often lacked a theoretical base for conceptualizing physician behavior change (50, 52). Applying theory in implementation research design has numerous advantages: it provides a process to guide the development, delivery, and evaluation of interventions, a generalizable framework within which to represent the dimensions that implementation studies address, and a way to identify and explore potential causal mechanisms for physician behavior change (52, 55, 56). Growing evidence also suggests that interventions that are explicitly grounded in a theoretical bases are more effective than those without, and that strategies that combine multiple theories and concepts have larger effects (57, 58). A comparable study that aimed to increase HVP vaccinations in a US pediatric primary care setting also followed a theory-informed development process to identify barriers and facilitators of practice for healthcare providers, designate target behaviors, and select strategies behavior change strategies.

Tailoring of a change intervention, by integrating pre-identified barriers and practice context characteristics, and the use of behavior change theory, are key processes more likely to improve professional practice than dissemination of guidelines and education materials alone (36, 59). Implementation barriers were determined with direct input from targeted intervention participants in the 2022 survey of BC pediatricians. Although IS models and protocols offer guidance to create interventions for facilitating practice change (38, 41, 60), the development process for this particular intervention did not follow a traditional sequence of planning.

Acknowledging CME is the most popular and well-received method of clinical upskilling for physicians in BC, the team pre-determined that at least one educational delivery mode would be essential, and the MOC credit offered an added incentive. The first task then was creating the intervention's desired and expected practice changes grounded on CME content curated by the team's developmental pediatric experts and community pediatrician based on clinical guidelines (9). This is a novel departure from most continuing professional development activities for physicians, which do not generally promote clinical behavior change (61). The change objectives were deemed necessary for participants to be able to practice in accordance with current CP diagnosis guidelines. Next, the IS expert and graduate trainee selected BCW mechanisms that were integrated into the intervention workshops to enable participants to reach the change objectives based on the identified TDF barriers. This approach greatly enhanced the efficiency of study progress given the limited research time within the team leads' existing clinical workload. Originally the team had considered an online education module, allowing for flexibility for pediatricians because of its self-paced and asynchronous nature. However, this strategy required intensive labor to create the module, a long timeline for CME accreditation approval, and nearly the entire study budget. Because of the low rating by the team on both practicability and affordability, it was removed as an option. Ultimately, the team deviated from the traditional sequence of intervention development (62) to embrace local considerations and team capacity.

Generalizability of such an intervention promoting practice change is high in other provinces and jurisdictions where there are pediatric care providers working in community settings. Ultimately the intervention was designed to support any pediatric care provider who is responsible for assessment and diagnosis of a common childhood motor disability like CP. The intervention can also be applied in other settings with different detection pathways if target participants can be clearly identified based on who has been designated with the responsibility of diagnosis. Incorporating IS frameworks and explicit behavior change mechanisms into CME also provides great potential for initiating practice change in provinces and countries where CME continues to be the accepted dominant format by physicians for professional development. However, CME development and delivery require significant budget, time, and labor. In addition, CME topics and offerings are typically identified by special interest groups or driven by faculty interest and available resources, rather than based on physician needs assessments (63).

Limitations of this study design include not assessing the intervention co-design process and participants' readiness for change. The BC pediatric workforce has been under strain in recent years, related to managing the COVID-19 pandemic, the seasonal surges of respiratory illness in children, major shifts in the provision of provincial child and youth services, and a growing population. Pediatricians in remote areas or who support under-served communities carry particularly heavy workloads, often as the sole provider covering large geographic areas that involve significant call requirements (64). Although the intervention was offered for all pediatricians, regardless of where they were situated in BC, those based in rural and remote areas were less likely able to participate in-person workshops that included the benefits such as dinner or face-to-face networking with facilitators and other participants. Comparison of participant experiences between the online and in-person formats was not included in the current evaluation plan.

Not all pediatricians will have capacity to participate in the intervention or identify with the subject matter of CP diagnosis as a priority. Interest will also vary depending on how frequently they have seen children who fall into this category of children without early detectable risk factors. All study participants volunteered to take part in the intervention can be an indication of prior interest or exposure in the subject matter of CP. For these reasons, intervention results cannot be extended to conclude the same mechanisms and strategies will be effective on the wider population of pediatricians.

All theoretical frameworks used in the intervention design process are focused on understanding and addressing individual behavior change. Although the TDF does include one domain on Environmental Context and Resources, it lacks specificity and limits understanding of the interplay of behavior under organization and systems-level contexts. A noted challenge during intervention development was designing the appropriate data collection tools that balanced the requirement for measuring change outcomes with the risk of deterring participation in the evaluation component, which was optional for participants receiving the intervention. As such, expected issues related to intervention delivery include lower survey response rates, recruitment of pediatricians, and the unpredictability of potential public health crises that may arise during study implementation. Lastly, evaluation data rely on participant self-reporting of practice behaviors that may also be affected by social desirability bias. Beyond practice changes at the provider level, investigations into objective clinical outcomes such as a decrease in age of diagnosis or increase of referrals for CP-specific early intervention are recommended for long-term follow-up.

## 5 Conclusion

To facilitate best practices in early diagnosis of CP in community settings, an interdisciplinary team in BC developed a tailored, theory-informed KT intervention for community pediatricians to address identified implementation barriers. Community pediatricians play a significant role for children at risk of CP as a first point of contact, particularly for those without early detectable risk factors and were referred for investigations by a primary care provider. The intervention design strengthens traditional CME by incorporating IS theoretical frameworks to change pediatricans' practice behaviors, target participant assessment of local barriers and facilitators, and evidence review of change strategies. Using select BCW mechanisms such as Education, Training, Modeling, Persuasion, and Enablement, target participants are expected to adopt six desired change outcomes necessary for best practice aligned with current evidence-based guidelines for CP diagnosis. These changes include three practice behaviors of promptly performing required assessments after CP warning signs are detected, communicating the CP diagnosis to parents and caregivers as per a recommended framework, and referring the child for early intervention services and supports. By providing a diagnosis as soon as possible, children can maximize gains from CP-specific early intervention and receive the necessary supports and services they deserve. Next steps of this study will be to observe and measure if participants have adopted the intended objectives and intervention acceptability after implementation.

## Data Availability

The original contributions presented in the study are included in the article/Supplementary material, further inquiries can be directed to the corresponding author/s.
