# Supplementary material for: Development of an evidence-based knowledge translation intervention to promote behavioral change in cerebral palsy diagnosis
Source: Front Public Health. 2026 Mar 16;14:1753404. doi: 10.3389/fpubh.2026.1753404 (PMC13033759; doi:10.3389/fpubh.2026.1753404)
Supplement: Supplementary file 1 [file Supplementary_file_1.docx]

**Part 1 - We recognize that some of you will have experience with children and youth with cerebral palsy and others are subspecialists who may have only occasional contact with children with cerebral palsy. Our first series of questions will help us understand your experience with children and youth with cerebral palsy and your practice needs.**

Please answer the following questions about your practice and experience.

1. In what year did you complete your residency or subspecialty training? [Drop-down menu with years]
2. Which of the following best describes your area(s) of work (select all that apply)?

- Complex Care
- Critical Care
- Developmental Pediatrics
- Emergency Medicine
- General Pediatrics
- Genetics
- Neonatology
- Neurology
- Other: _____________

1. Which best describes your practice setting?

- Metro (population over 190,001)
- Urban/rural (population between 40,001-190,000)
- Rural (population between 10,001 – 40,000)
- Remote (population between 0 and 10,000)

1. In which health region(s) of BC do you work? (Select all that apply). Note: This question refers to the geographic region, not the specific health authority that may employ you.

- Fraser Health
- Interior Health
- Island Health
- Northern Health
- Vancouver Coastal Health

1. What type of clinical setting do you work in? (check all that apply)

- Hospital: inpatient
- Hospital: outpatient
- Community
- Other: ______________________

1. Please estimate the number of children per month you see in your practice who have cerebral palsy.

- I do not see children with CP
- 0-1 (in some months I see 1 and in other months I see none)
- 2-3
- 4-6
- 7-9
- 10+

*If answer is “I do not see children with CP”, message will appear: “You have identified that you do not see children with cerebral palsy in your practice. We have some final questions related to your interest in learning about working with children with cerebral palsy.” Participants will answer questions 7-8,19-22.*

**Part 2 - We would now like to start identifying your learning needs by asking about your understanding of cerebral palsy.**

1. Please answer the following True and False questions about cerebral palsy:

- Abnormal brain imaging is required for a diagnosis of cerebral palsy.
- Cerebral palsy is an umbrella term that is not defined by etiology.
- Children with cerebral palsy deteriorate over time.
- Of all children with cerebral palsy, 40% are born prematurely and 60% are born at term.
- Predicting severity of cerebral palsy is most accurate after age 2 years.
- A diagnosis of cerebral palsy can only be made when the cause of the child’s motor impairment is known.
- Evidence supports the early diagnosis of cerebral palsy.
- A diagnosis of CP cannot be made before the age of 12 months
- A diagnosis of CP should be delayed since it increases the mental and emotional stress for caregivers

1. Which of the following underlying causes of motor impairment can result in cerebral palsy? [select all that apply]

- Periventricular leukomalacia
- Perinatal brain injury
- Spinal cord injury
- Acquired brain injury during the first 2 years of life
- Chromosomal abnormality
- Genetic causes
- Metabolic condition
- Muscular origin
- Congenital cerebral malformation
- Postnatal infection (meningitis/encephalitis)
- Intrauterine infection
- Maternal thyroid deficiency
- Unknown etiology

**Part 3 - The following questions are related to your current practice with children with cerebral palsy. Your answers to these questions will help inform the educational opportunities we will create.**

1. Are you currently diagnosing children with cerebral palsy in your practice? Yes/No

*Questions 8-10 will be displayed if respondent answered yes*

1. How often do you diagnosis children with CP per year? [open text]
2. Generally, at what age do you typically provide a diagnosis of CP?

- 0-2 years
- 3-5 years
- 6-9 years
- 10+ years

1. Do you provide resources to the family after the diagnosis? (Select all that apply)

- Yes, information about what cerebral palsy is
- Yes, information about what the future may involve for their child
- Yes, information or referrals to support services and funding
- No; I would like to but I’m unaware of what’s available
- No; I don’t find resources to be helpful

1. Please rate your agreement with the following statement: *Providing a diagnosis of cerebral palsy is important*

- I strongly agree
- I agree
- I am ambivalent
- I disagree
- I strongly disagree

1. What type of clinician(s) do you think should be responsible for making a diagnosis of CP? Select all that apply

- General practitioner
- General practitioner, with support from pediatrician/developmental pediatrician/neurologist as needed
- Pediatrician
- Pediatrician, with support from developmental pediatrician/neurologist as needed
- Pediatrician, with input from physiotherapists or occupational therapists
- Developmental pediatrician
- Neurologist
- Orthopaedic surgeon
- Other: __________________________

1. Are there **knowledge, skills**, **or confidence** factors that impact your providing an early diagnosis of CP? (Select all that apply)

- I do not have enough knowledge about how to make a diagnosis of CP
- I feel uncertain making a diagnosis for children at a young age (e.g., under 2 years)
- I am uncertain if the motor delay is due to other diagnoses
- I prefer to monitor to determine if motor delay will improve over time
- I have difficulty recognizing early motor type, topography, and severity of CP
- I do not feel comfortable diagnosing when I am unable to classify a GMFCS (Gross Motor Function Classification System) level
- I am concerned about making a false positive diagnosis
- I don’t feel comfortable communicating this diagnosis to a family
- I am not sure of the next steps after making a diagnosis
- I do not know what supports are available for the child and family
- Other (specify): __________________________
- Not applicable

1. Children with CP have different risk factors and clinical presentations that may influence diagnosis. Please rate your confidence in making a diagnosis in the following situations (anchors on 1=not at all and 5=very confident).

- Born premature, delayed development, spasticity
- Born premature, delayed development, spasticity, periventricular leukomalacia (PVL) on imaging
- Born premature, delayed development, low tone
- Born term, delayed development, increased tone
- Born term, delayed development, low tone

1. Are there **environmental or systems** factors, such as your practice setting, resources, funding, or policies, that impact your providing an early diagnosis of CP? (Select all that apply)
   - Length of appointment time (e.g., not enough time required to do an assessment)
   - Patient waitlist length (e.g., long waitlist prevents assessing a child in a timely manner)
   - Access to therapists (OT or PT) in my community to help inform the CP diagnostic process
   - No systems in place in my practice to triage cases
   - Lack of imaging services to support the diagnosis
   - Delayed referrals to specialists due to child not having a family doctor
   - No professional support or colleagues with expertise/experience to review cases/consult
   - My peers and colleagues currently are not providing CP diagnoses
   - Time required or lack of time to learn about diagnosing CP
   - Access to education/training on CP diagnosis
   - Other: _____________
   - Not applicable
2. Are there **other factors** that impact your providing a diagnosis of CP? (Select all that apply)

- I prefer to refer to a specific specialist for diagnosis; I do not think it is my role to provide the diagnosis
- I feel the family wants a diagnosis from a neurologist, developmental pediatrician or other specialist
- It is not important to have a CP diagnosis in order to treat functionally
- I don’t think families are ready for the diagnosis and the burden it will place on them
- I don’t think it is important to provide a diagnosis of CP since it will not change the child’s outcome or if the child already has access to therapy or supports
- I prefer not to use labels like CP
- Other: ______________
- Not applicable

1. Are you familiar with or trained to use any of the following clinical assessments? [select all that apply]
   - Hammersmith Infant Neurological Exam (HINE)
   - General Movements Assessment (GMA)
   - Test of Infant Motor Performance (TIMP)
   - I am familiar with or trained to use this other clinical assessment tool(s) to diagnose CP: ______________
   - I am not familiar with or trained to use any clinical assessments to diagnose CP
2. Which of the following clinical assessments are you actively using in your practice? [select all that apply] *Question will only be displayed if respondent* ***does not choose*** *“I am not familiar with...”*
   - Hammersmith Infant Neurological Exam (HINE)
   - General Movements Assessment (GMA)
   - Test of Infant Motor Performance (TIMP)
   - I am using this other clinical assessment tool(s) to diagnose CP: ______________
3. **To increase your knowledge and skills** specific to the needs of children with CP, please tell us the **top five topics** **about which** **you are most interested in** (select up to 5 from the entire list):

Diagnosis

- - Completing the required assessments related to tone and cerebral palsy (e.g., physical exam or HINE)
  - Criteria for diagnosing a child with CP using a care pathway
  - The different types of CP
  - Determining a child’s classification levels - e.g., GMFCS, Manual Ability Classification System (MACS), Communication Function Classification System (CFCS)
  - Testing to determine the etiological cause of cerebral palsy
  - Assessing other areas of development in the context of motor impairment
  - How to communicate a diagnosis of CP

Treatment

- - Identifying children that require hip surveillance
  - Assessing and managing tone
  - Managing seizures in children with CP
  - Diagnosing and managing sleep problems in children with CP
  - Managing nutrition and feeding problems in children with CP
  - Assessing and managing pain in children with CP
  - Next steps after making a diagnosis of CP (e.g., early intervention, medical management, community referrals and other considerations)
  - Prognostication of future function after diagnosing CP
  - Advising families on complementary and alternative medicines

Resources

- - Providing family education on preventative healthcare (e.g., special immunizations, dental care)
  - Providing families with resources (e.g., information about CP, referrals to support services, financial resources)
- Other:________________

1. What type of education/training best suits your learning needs? (Select all that apply)

- Virtual on-demand webinars
- Virtual live webinars
- In-person education/training opportunities
- Self-paced online learning modules
- Physician-to-physician consultation with a specialist, such as a developmental pediatrician
- A community of practice (peers coming together to support learning needs)
- Mentorship within my discipline
- Other ideas or suggestions: ___________________

1. What would make it most feasible for you to take advantage of these education/training opportunities? [Open text]
