# Supplementary material for: Development of an evidence-based knowledge translation intervention to promote behavioral change in cerebral palsy diagnosis
Source: Front Public Health. 2026 Mar 16;14:1753404. doi: 10.3389/fpubh.2026.1753404 (PMC13033759; doi:10.3389/fpubh.2026.1753404)
Supplement: Supplementary file 2 [file Data_Sheet_1.PDF]

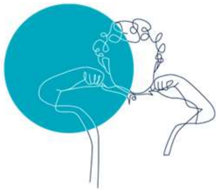

Cerebral Palsy Diagnosis in Community Pediatrics: Using the New Clinical Pathway

Prince George, BC
September 11, 2024

Dr. Ram Mishaal
Developmental Pediatrician
Sunny Hill Health Centre, BC Children's Hospital

Clinical Assistant Professor
Division of Developmental Pediatrics
Department of Pediatrics
UBC Faculty of Medicine

1

Learning Objectives

1. List the evidence and benefits of an early diagnosis of CP
2. Identify the key clinical red flags for CP in children
3. Implement the necessary assessments for CP diagnosis
4. Apply assessment findings to determine CP diagnosis in children
5. Communicate a CP diagnosis with parents and caregivers
6. Utilize resources and supports for CP in children

2

Cerebral Palsy – Definition

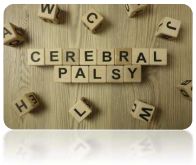

"Cerebral palsy is a group of permanent disorders of the development of movement and posture, causing activity limitation, that are attributed to non-progressive disturbances that occurred in the developing fetal or infant brain."

— Rosenbaum et al., 2007

3

Cerebral Palsy – Etiology

Brain injury at different stages:
- Prenatal
- Perinatal
- Postnatal

4

Early diagnosis of CP – Why?

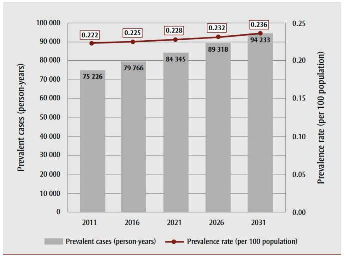

- Amankwah et al., 2020

- Number of newly diagnosed CP cases: from ~1800 in 2011 to ~2200 in 2031
- Number of people living with CP: expected to increase from >75,000 in 2011 to >94,000 in 2031

5

Early diagnosis of CP – Why?

For children:
- Inform the prognostic care plan
- Expedites early intervention to maximize neuroplasticity
- Prevents related complications

For parents and caregivers:
- Reduces distress of uncertainty, improves mental health and facilitates future planning

For the family as a unit :
- Fosters greater acceptance, coping, and adaptation to the new diagnosis

6

Early diagnosis of CP – What we know in BC

- Canadian average age of diagnosis: ~19.5 months[1]
- BC average age of diagnosis: ~25 months[2]

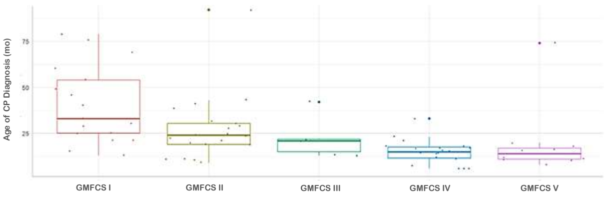

1. Boychuck et al., 2019,
2. 2021 review of the Canadian Cerebral Palsy Registry by Taylor McIntosh, UBC MD Candidate 2024

7

Community pediatrician are key to diagnosis

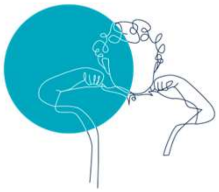

Particularly for lower-risk term-born infants without early discernible indicators of cerebral palsy

8

Red flags of CP in infants

1
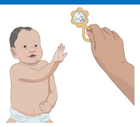
Demonstrates a hand preference before 12m

2
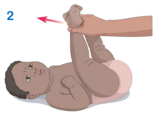
Demonstrates stiffness or tightness in the legs

3
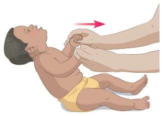
Demonstrates a persistent head lag beyond 4m of age

4
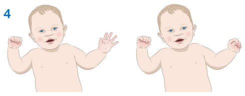
Keeps their hands fisted (closed/clenched) after the age of 4m

Recommendations from: BC Cerebral Palsy Advisory Committee 2017-2021 and The PROMPT Group 2019

9

Red flags of CP in infants

5
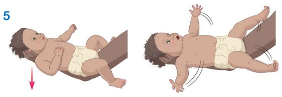
Demonstrates persistent primitive reflexes, including startle (Moro) reflex beyond 6mo of age, or "Fencer" (ATNR) beyond 4m of age

6
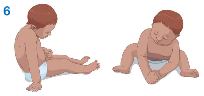
Not able to sit without support beyond 9m of age

7
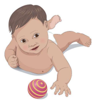
Demonstrates consistent asymmetry of posture and movement after the age of 4m

8
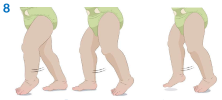
Demonstrates consistent toe-walking or asymmetric-walking beyond 12m of age

9 Unable to walk by 18m of age

Recommendations from: BC Cerebral Palsy Advisory Committee 2017-2021 and The PROMPT Group 2019

10

How is CP diagnosed?

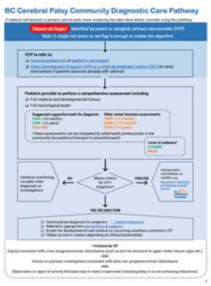

Introducing a new care pathway to support clinical decision-making in diagnosis of CP

11

How is CP diagnosed?

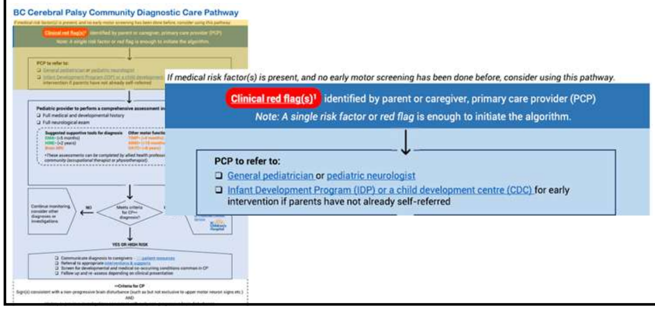

12

How is CP diagnosed?

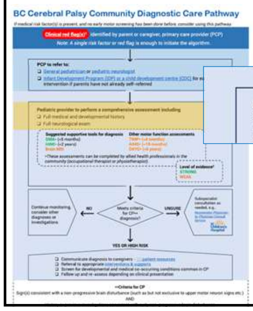

Pediatric provider to perform a comprehensive assessment including
- Full medical and developmental history
- Full neurological exam

13

How is CP diagnosed?

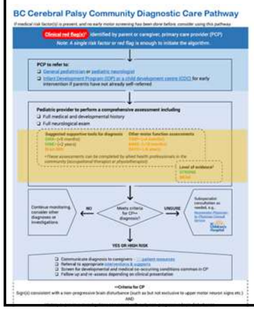

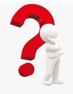

Don't I always need an MRI to make a diagnosis?

Answer: no!

Recommendations for MRI when…

- History does not match clinical findings
- Atypical CP (e.g., hypotonic)
- History of developmental regression or other history suggestive of progressive brain disturbance

14

How is CP diagnosed?

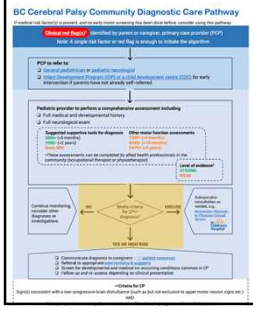

Do findings meet criteria for CP diagnosis?

Sign(s) consistent with a non-progressive brain disturbance (such as but not exclusive to upper motor neuron signs etc.)

AND

History or investigations consistent with early non-progressive brain disturbance

AND

Observation or report of activity limitation due to motor impairment (including delay in or not achieving milestones)

15

How is CP diagnosed?

- ✓ A group of permanent disorders of the development of movement and posture
- ✓ attributed to non-progressive disturbances that occurred in the developing fetal or infant brain
- ✓ causes activity limitation

Do findings meet criteria for CP diagnosis?

Sign(s) consistent with a non-progressive brain disturbance (such as but not exclusive to upper motor neuron signs etc.)

AND

History or investigations consistent with early non-progressive brain disturbance

AND

Observation or report of activity limitation due to motor impairment (including delay in or not achieving milestones)

16

Classifying CP

Types of CP based on Movement and topography[7]

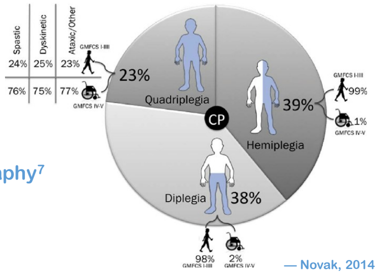

— Novak, 2014

17

Levels in CP

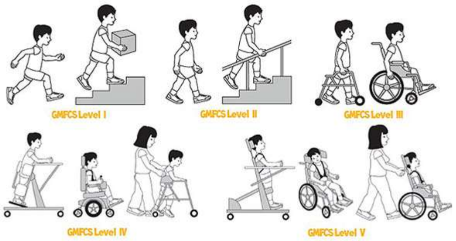

— GMFCS descriptors: Palisano et al. (1997)
© Bill Reid, Kate Willoughby, Adrienne Harvey and Kerr Graham
The Royal Children's Hospital Melbourne

18

CASE STUDY

If you are missing a copy let us know!

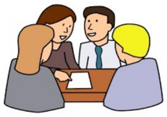

19

Preparing for a conversation

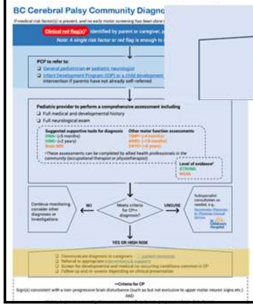

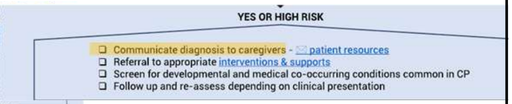

Types of information and knowledge needed by parents[5]

1. The diagnosis
2. Interventions
3. Daily caregiving
4. Equipment
5. Supports
6. How to explain the disability to others
7. The effects on the family
8. The future

— Adapted SPIKES protocol by Novak et al

20

Communicating the diagnosis

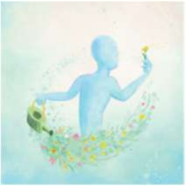

Hope and realism: hope springs eternal[6]
- Kaye et al., 2020

Key ingredients for a supportive diagnosis conversation[5]:

Well-planned
+
Compassion

— Adapted SPIKES protocol, Novak et al, 2019

21

Post-diagnosis best practice

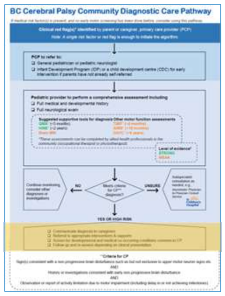

Screen for developmental and medical co-occurring conditions in CP

- Vision and hearing impairments
- Cognitive, learning, and attention impairments
- Seizures
- Musculoskeletal concerns
- Pain
- Sleep
- Feeding and swallowing
- Speech and language delay
- Bowel and Bladder

22

Co-occurring conditions with CP[7]

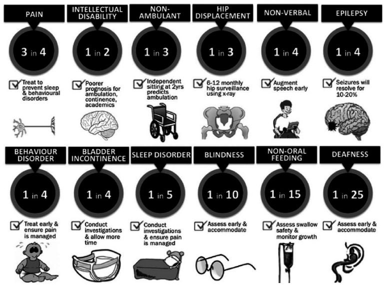

— Novak, 2014

23

Post-diagnosis best practice

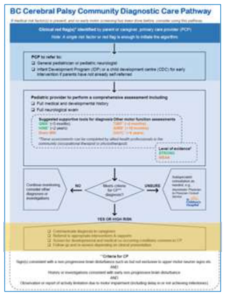

Referrals to early intervention and supports

- IDP/CDC (specify OT/PT/SLP/Rec Therapy)
- BC Hip Surveillance Program
- At Home Program
- Disability Tax Credit if appropriate
- Parking pass

24

Further Learning and Resources

Sunny Hill Neuromotor Physician to Physician consult

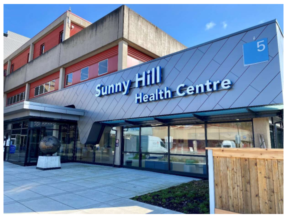

- Virtual consults available with developmental pediatricians
- 15-20 min appointments available
- Download booking form online – Sunny Hill Website

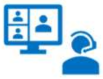

25

Further Learning and Resources

Hammersmith Infant Neurological Exam

https://hollandbloorview.ca/our-services/programs-services/neuromotor-services/hammersmith-infant-neurological-examination-hine

Pathways™

Request an account and find all the resources mentioned in one hub
https://pathwaysbc.ca/login

UBC CPD
https://ubccpd.ca/cp-resources

26

Your role as a community provider

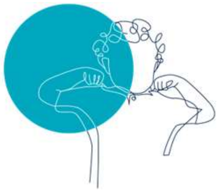

Community providers have a pivotal role in the diagnosis of CP.

Find and get to know PT/OTs allies in your communities!

27
